# Prodrugs of Thyrotropin-Releasing Hormone and Related Peptides as Central Nervous System Agents

**DOI:** 10.3390/molecules14020633

**Published:** 2009-02-06

**Authors:** Katalin Prokai-Tatrai, Laszlo Prokai

**Affiliations:** 1 Department of Pharmacology & Neuroscience, University of North Texas Health Science Center, Fort Worth, TX, USA; 2 Department of Molecular Biology & Immunology, University of North Texas Health Science Center, Fort Worth, TX, USA; E-mail: lprokai@hsc.unt.edu (L.P.)

**Keywords:** Neuropeptide, CNS-delivery, TRH, Analogue, Prodrug, Prodrug-amenable analogue.

## Abstract

Prodrug design for brain delivery of small- and medium-sized neuropeptides was reviewed, focusing on thyrotropin-releasing hormone and structurally related peptides as examples. We have summarized our most important advances in methodology, as well as assessed the benefits and limitations of bioreversible chemical manipulation techniques to achieve targeting of the parent molecules into the central nervous system. The value of prodrug-amenable analogues as potential drug-like central nervous systems agents was highlighted.

## 1. Introduction

Drugs face a formidable obstacle in reaching the central nervous systems (CNS) due to the existence and specific properties of the blood-brain barrier (BBB) that is a vital element in the regulation of the delicate environment of the brain and the spinal cord [1]. Capillary endothelial cells display tight junctions in the brain, which eliminates any paracellular pathways into the inner milieu of the brain. To most solutes, the BBB essentially behaves like a continuous lipid bilayer and, thus, exhibits low permeability to hydrophilic substances (ions and polar compounds) that do not have specific transport mechanisms. Consequently, hydrophilic molecules such as peptides potentially useful as CNS-agents cannot cross the BBB in pharmacologically significant amounts. On the other hand, lipophilic substances are generally transferred across the BBB by passive transport. Highly active enzymes also represent a metabolic component that contributes to the homeostatic balance regulated by the BBB [2]. Most peptides are metabolically unstable because they are degraded by peptidases, e.g., in the cytosolic endothelial space, the luminal surface of the endothelial cells, cerebral pericytes, and/or synaptic regions juxtaposed to the brain microvessels, even if they have certain permeability. The transmembrane glycoprotein known as P-glycoprotein (P-gp) is also expressed in the BBB [3]. P-gp has been shown to operate as an active efflux system, and it generally transports back into the blood a variety of lipophilic molecules that enter the endothelial cells. P-gp should be considered a significant and functional part of the BBB [4], as it has been shown that P-gp knock-out mice show enhanced sensitivity to circulating drugs and toxins. Several other transporters may also contribute to the exclusion of certain drugs from the CNS, because they work in the direction of interstitial fluid to endothelial cell and/or endothelial cell to blood [5]. 

To overcome the BBB as an obstacle for the pharmacotherapy of the CNS, drug delivery via both invasive and non-invasive methods has been a longstanding and actively pursued endeavor [6]. Invasive strategies go around the BBB or alter/modify it to provide entry into the interstitial fluid of the brain or spinal cord (e.g., via lumbar punctures or by reversible BBB disruption) [7]. These types of procedures are only justified for life-threatening CNS maladies such as brain cancer; however, these costly surgical routes are not preferred for less dramatic illnesses. Non-invasive techniques exploit various transport processes that exist in the brain capillary endothelium to ferry therapeutic agents into the CNS after systemic administration. However, the plausible method of designing drugs that exploit carrier-mediated uptake mechanisms present in the endothelial cells of the CNS vasculature for specific biomolecules suffers from a critical kinetic feature of these systems, since they are often of low capacity, although of high affinity [8]. Additionally, current knowledge about the structural requirement of a drug capable of fully utilizing these specific transporters is limited. Efforts to exploit biological carriers have, therefore, concentrated on auxiliary transport systems with various degrees of success [9,10,11,^12^,^13^].

For CNS-delivery of small- to medium-sized neuropeptides and their analogues/mimetics, we advocate the prodrug approach. This versatile, non-invasive chemical manipulation technique relies on the bioreversible alteration of the target peptide to produce its inactive (bio)precursor (i.e., without intrinsic activity) having improved physicochemical characteristics to CNS-transport compared to those of the parent agent. The term ”prodrug” was first introduced by Albert in the late 1950’s [14] to define pharmacologically inactive chemical derivatives that could be used to alter the physicochemical properties of drugs in a transient manner to increase their usefulness and/or to decrease associated toxicity. In the classical sense, prodrugs are aimed at reaching the CNS by diffusion (passive transport), although pro-moieties that rely on carrier-mediated (active) transport have also been reported [15]. Passive transport through the BBB is controlled by several physicochemical parameters such as size (more exactly molecular volume), charge and hydrogen-bonding (donor or acceptor) capacity [16,17,18]; nevertheless, lipophilicity (expressed as the logarithm of the *n*-octanol/water partition coefficient, logP) is generally considered the most important indicator for BBB penetration [19,20]. A logP of around 2 (i.e., 100-times higher affinity to the lipid-mimicking n-octanol than to water) is believed to be an optimal value for CNS-delivery [21]. However, as mentioned above, efflux mechanisms [22] operating in the BBB must be also considered in prodrug design, because they can remove the prodrug from the brain even in case of a robust influx [23,24] resulting in poor CNS-retention and short biological half-life. Once the prodrug crossed the BBB, conversion by “post barrier” enzyme(s) [25] is utilized to regenerate the parent peptide. 

Bioreversible alteration of poorly CNS-available peptides involves chemical derivatization of the parent peptide by taking advantage of the inherently present functional group(s) of the peptide chain. Those chemical ‘handles” may be the amino- and carboxyl-termini or the side chain’s functional group (e.g., amino-, hydroxyl-, or carboxyl group). The appropriate transient masking of these polar groups will also decrease hydrogen-bonding capacity and render the prodrug neutral at physiological pH to promote passive transport through the BBB. Additionally, precise placement and choice of these cleavable “pro-moieties” can also provide protection against exo- and endopeptidases. In the blood, many small peptides with free *N*- and *C*-termini are degraded primarily by exopeptidases usually within a few minutes. Protection against peptidase recognition is, therefore, one of the critical aspects of peptide-based prodrug design, because even if these produgs can cross the BBB, they can only sustain adequate concentrations in the brain, if their blood concentration is maintained at sufficiently high levels by preventing their systemic degradation.

Brain-targeting prodrugs [26] are extensions of simple prodrugs in terms of having a specific pro-moiety whose major function is to promote access and retention in the brain. This may be achieved by *in situ* metabolic conversion of a non-ionic pro-moiety (e.g., 1,4-dihydropyridine) to an ionic (e.g. pyridinium) group as an intermediate before the release of the active agent [27]. In neuropeptide-based drug design, the real challenge often is, however, to meet the requirement of creating analogues/mimetics that preserve the intrinsic CNS effect(s) of the native peptide while abolishing its undesired systemic (e.g., endocrine) effects and metabolic susceptibility toward peptidases. In the most elegant approach, the analogue/mimetic is designed “prodrug amenable,” which permits transient chemical manipulation and allows for prodrug formation without the covalent attachment of any auxiliary pro-moiety (or pro-moieties) to facilitate transport across the BBB. 

**Figure 1:**
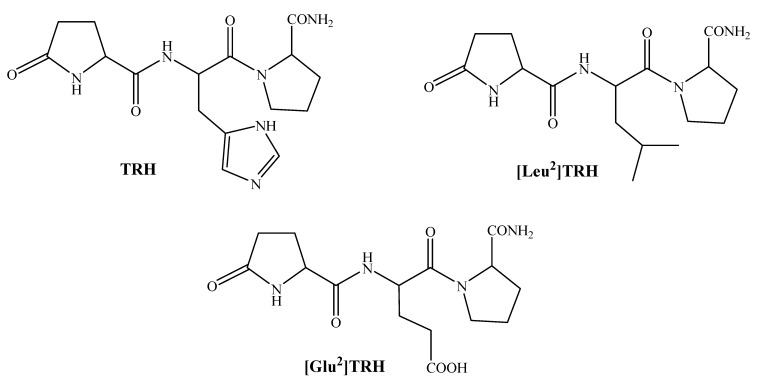
Chemical structure of TRH and its analogues used in our studies.

For the development of brain-targeting prodrug strategies involving neuropeptides we have studied most extensively thyrotropin-releasing hormone (TRH, pGlu-His-Pro-NH_2_) and its analogues (Figure 1) as models for neuropeptides with poor access to the CNS [28]. In this review, we summarize the most important developments our laboratory has achieved in this field in the last 10 years. TRH and structurally related endogenous peptides (Figure 1) have been considered lead compounds for developing useful CNS agents [29]. This small peptide was the first hypothalamic releasing factor characterized, establishing the fundamental proof for the existence of a neuroendocrine regulation of pituitary functions by hypothalamic neuronal structures [30,31]. A variety of behavioral effects are induced by its peripheral and central application [32]. Therefore, it has been implicated in the management of various neurological and neuropsychiatric disorders such as depression, epilepsy, brain injury, Alzheimer’s disease and schizophrenia, as well as stimulation of spinal-cord motorneurons. TRH has been successfully used for treating children with neurological disorders including epilepsy conditions intractable to anticonvulsants and adrenocorticotrophic hormone, mostly in Japan [33]. The best-known neuropharmacological effect of TRH is its analeptic action [34,35], which is frequently exploited for testing TRH-based compounds in early-phase development [29]. High doses administered peripherally and lower doses administered into specific brain regions, have been shown to significantly reduce pentobarbital-induced sleeping time in rats, rabbits and monkeys. The analeptic effects of TRH appear to be mediated by a cholinergic mechanism [36,37].

Studies in rodents and humans have shown that this tripeptide has a very short (6–10 min) half-life in plasma [38,39]. The rapid degradation in this medium is mainly due to the action of pyroglutamyl aminopeptidases [40]. Additionally, this highly hydrophilic molecule (logP = –2.46) also lacks its own active BBB transport system [41]; thus, high doses of the peptide unavoidably manifesting a profound and undesired hormonal (endocrine) effect would be required for CNS application. Therefore, dissociation of endocrine and CNS actions of TRH is a crucial issue in designing TRH analogues and mimetics for neuropharmacotherapy. Altogether, efforts have been focused on designing metabolically stable and centrally active analogues/mimetics. 

## 2. TRH and Related Peptides as CNS Agents

### 2.1. [Leu^2^]TRH prodrugs

**Figure 2:**
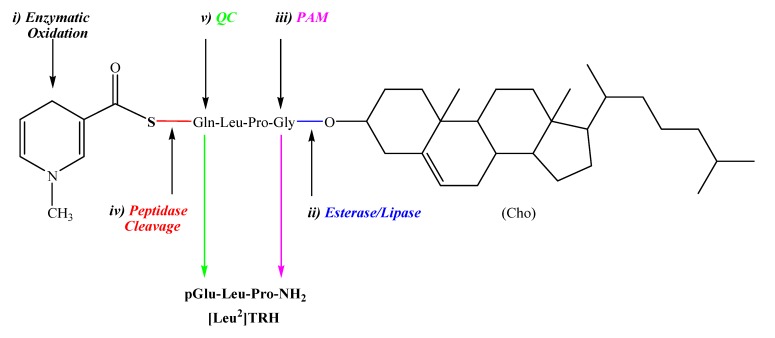
Schematic illustration of the prodrug concept developed for the brain delivery of [Leu^2^]TRH utilizing Gln-Leu-Pro-Gly progenitor sequence and enzymes (*i* to *iv* ) inherently present in the brain.

A centrally selective natural analogue of TRH is [Leu^2^]TRH (pGlu-Leu-Pro-NH_2_, Figure 1) [42], in which the central His of TRH is replaced with Leu. Compared to TRH, [Leu^2^]TRH shows an approximately 2.5-fold increase in typical TRH-associated analeptic effect and 50-fold decrease in promoting TSH release [43]. Therefore, we selected this tripeptide for substantiating our brain-targeting prodrug approach [25]. Nevertheless, [Leu^2^]TRH possesses a profound peptide character such as poor lipophilicity (logP = –1.67) that prohibits significant transport across the BBB. The amino- and carboxy-termini of [Leu^2^]TRH are not suitable for direct bioreversible derivatization to create a lipophilic prodrug by our approach; however, we envisioned that (by the analogy of TRH biosynthesis [44]) Gln-Leu-Pro-Gly would be a suitable progenitor sequence for [Leu^2^]TRH. Specifically, it was expected that peptidyl-a-hydroxy-glycine (PAM, [45]) would convert the carboxy-terminal Gly to Pro-NH_2_ and glutaminyl cyclase (QC [46]) would eventually produce pGlu from Gln by using this progenitor sequence. A “transport moiety” (1,4-dihydrotrigonellyl, Dht, residue) [26] for the prodrug creation was attached through strategically selected [47,48] scissile linkers (**S**) to the amino-terminus of the Gln-Leu-Pro-Gly progenitor sequence [25], as shown in Figure 2. Dht is expected to be oxidized to pyridinium (trigonellyl, Trig; the residue of *N*-methylnicotinic acid) in the brain analogously to that of NAD(P)H to NADP^+^ and, therefore, the resultant “oxidized prodrug” is captured inside the brain due to its ionic nature. To furnish lipophilicity to the prodrug for BBB transport, its carboxy-terminus was esterified with highly lipophilic alcohols such as cholesterol (Cho). The idea behind this design was that [Leu^2^]TRH is eventually formed in the brain after a well-orchestrated sequence of enzymatic processes (*i* to *v*, Figure 2). 

A series of *in vitro* and *in vivo* experiments were employed in our proof-of-concept studies due to the complexity of the molecular architecture and the necessity of a multistep bioactivation process leading to the liberation of [Leu^2^]TRH [25]. We thoroughly investigated the cascade of enzymatic reactions responsible for the release of the core peptide by using appropriate putative metabolites. One of the most important steps is the Pro-NH_2_ formation on the carboxy-terminus. *In vitro* studies showed that this process was significant only in the brain. In blood and liver, the primary degradation produced an inactive metabolite having Pro-OH as the carboxy-terminal residue and moreover, the metabolism of the “oxidized” prodrug was much faster in the brain than in the blood and liver; thus, systemic formation of [Leu^2^]TRH was practically prevented. The peptidolytic cleavage catalyzed by enzymes such as dipeptidyl dipeptidases (EC 3.4.14.2 and/or EC 3.4.14.5) [47,49] for **S**=Pro/Ala, or proline oligopeptidase (POP, EC 3.4.21.26) [48] when **S** is a dipeptidyl moiety (e.g.: Ala-Pro, Table 1) to form Gln-Leu-Pro-NH_2_ (Figure 2) and the subsequent rapid formation of [Leu^2^]TRH by QC without significant side-reactions were also unequivocally detected *in vitro* [25].

**Table 1:** Barbiturate-induced sleeping times in mice after the administration of [Leu^2^]TRH and its produgs having various spacers (**S**). Sleeping time decrease is expressed as percent of sleeping time for vehicle administration only (54.9 ±3.8 min). Ten min after i.v. injection of the test compounds at a dose of 15 µmol/kg body weight, pentobarbital (60 mg/kg, i.p.) was injected. Sleeping time was recorded from the onset of the loss of the righting reflex until the reflex was regained. The statistical significance of differences between groups was determined using analysis of variance (ANOVA) followed by *post hoc* Fisher’s PLSD test for multiple comparisons: *P < 0.05 vs vehicle control, **P <0.05 vs both [Leu^2^]TRH and vehicle.

Compound	% Decrease in Sleeping Time
[Leu^2^]TRH	17 ± 7*
Prodrug, **S**= Ala	30 ± 3*
Prodrug, **S**= Pro	47 ± 6**
Prodrug, **S**= Ala-Ala	32 ± 4*
Prodrug, **S**= Pro-Ala	56 ± 4**
Prodrug, **S**= Pro-Pro	55 ± 7**

*In vivo* substantiation of the design was done by monitoring the CNS-mediated analeptic effect, a convenient pharmacological paradigm for the evaluation of TRH-related peptides, to assess the increase in the access of the prodrugs to the CNS upon i.v. injection of the prodrugs having various **S** moieties attached to the amino-terminal Gln of the progenitor sequence. Antagonism on the barbiturate-induced anesthesia indicates the extent of the activation of cholinergic neurons [37] and, thus, the successful central delivery of [Leu^2^]TRH by the prodrugs. As shown by the results summarized in Table 1, the unmodified parent peptide, [Leu^2^]TRH, decreased the sleeping time only by 17 ± 7% compared to the vehicle control (propylene glycol/DMSO 2:1 v/v; sleeping time 54.9 ± 3.8 min), while the most significant (p< 0.05) analeptic effect was achieved with prodrugs having Pro residue in their **S** function. The data in Table 1 also highlight the critical influence of the peptidase-catalyzed **S**-Gln cleavage on the observed pharmacological effect. The crucial role of the scissile peptide linkers (**S**) in successful peptide delivery in to the brain by analogous prodrug design was also confirmed for a Leu-enkaphalin analogue [50,51]. 

### 2.2. [Glu^2^]TRH Prodrugs

The TRH-like tripeptide [Glu^2^]TRH (pGlu-Glu-Pro-NH_2_, Figure 1), although originally identified from rabbit prostate [52], has been shown to occur in the human brain [53]. Many pharmacological activities of this peptide are similar to those of TRH [54]; however, the beneficial effects after treatment with [Glu^2^]TRH are reportedly more robust or prolonged. These advantages are probably due to the increased resistance of this peptide to enzymes principally responsible for the rapid degradation of TRH *in vivo* [40]. In addition, [Glu^2^]TRH practically does not bind to TRH receptors and does not elevate triiodothyronine (T3) levels which has indicated that the peptide probably exerts pharmacological effects through binding to its own receptor in the CNS. 

**Figure 3:**
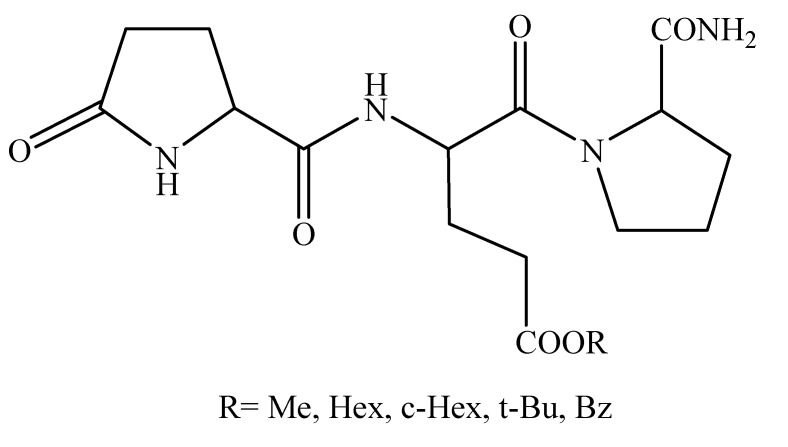
Structure of [Glu^2^]TRH prodrugs as potential CNS agents.

Due to the presence of the central Glu, this peptide is, however, predominantly ionized at physiological pH at the γ-carboxyl group of Glu, which would prevent pharmacologically significant amounts of peptide entering the brain by passive transport. Therefore, we carried out a bioreversible modification to afford prodrugs on this γ-COOH of the Glu residue to render the molecule neutral (non-ionizable) and, hence, amenable for diffusion across the BBB [55]. The synthesized esters (R = methyl: Me; Hex: *n*-hexyl; c-Hex: cyclohexyl; t-Bu: *tert*-butyl; Bz: benzyl) were first evaluated for their potential to interact with biological membranes by immobilized artificial membrane chromatography (IAMC) [56,57,58]. IAMC measures partitioning into monolayers of cell membrane-like phospholipids immobilized by covalent binding on silica particles and therefore, mimics membrane interactions better than partitioning in the isotropic *n*-octanol/water system (logP). The chromatographic capacity factor (k’_IAM_) for a compound obtained by the method is directly related to its partition coefficient between the aqueous phase and the chemically bonded membrane phase and, ultimately, to the K_m_ value representing its fluid-membrane partition coefficient. 

Table 2 shows that all ester prodrugs had increased membrane affinity compared to the unmodified parent peptide, and the hexyl ester (R = *n*-Hex) yielded the highest k’_IAM_ (16.0). *In vitro* stability studies in mouse brain homogenate (20%, w/v) revealed that the half-lives were around 20 min and 22 min for R = Me and Hex, respectively, 25 min for R = c-Hex, and 70 min for the sterically hindered ester (R = *t*-Bu). On the other hand, the aromatic benzyl ester (R = Bz) was quite stable in the tissue (t_½_ > 2 h) that rendered the compound practically useless as a prodrug. We utilized again the robust analeptic activity of [Glu^2^]TRH as an indicator of the CNS effect, although the potency of this compound as an analeptic agent is less than that of TRH [35]. When compared to an equimolar dose of the parent peptide (10 µmol/kg body weight, i.v), prodrugs having R=Me, Hex, c-Hex, respectively, showed a statistically significant (*P*<0.05) decrease in the sleeping time compared to that of unmodified parent peptide. The measured pharmacological effect appeared to correlate well with the *in vitro* metabolic stability of the prodrugs measured in mouse the brain homogenate. 

A slight influence of the increase in membrane affinity was also revealed upon comparing the analeptic response of the aliphatic methyl- and *n*-hexyl esters (Table 2). In all, esterification of the COOH in the side chain of the Glu residue with primary alcohols (the more lipophilic the better) afforded the most promising prodrugs for an efficient central delivery of [Glu^2^]TRH. Dose-response studies with the “best-performing” prodrug (R=Hex) also revealed that ED_50_ was around 2 µmol/kg body weight—a significant (10-fold) improvement in efficacy compared to that of the unmodified parent peptide. 

**Table 2:** IAMC capacity factors (k’_IAM_), *in vitro* half-lives (in 20% w/v mouse brain homogenate) and analeptic effects of test compounds (i.v. in mice at equimolar dose of 10 µmol/kg body weight when pentobarbital (i.p., 60 mg/kg body weight) was administered 10 min after the injection of the saline vehicle or test compounds) [55]. The k’_IAM_ value was calculated as (t_R_-t_0_)/t_0_, where t_R_ is the retention time for the compound and t_0_ is retention time of [Glu^2^]TRH used as a marker for dead volume. Statistically significant differences (ANOVA followed by post hoc Tukey test, *P*<0.05) from the control group (vehicle) and from both control group and Glu^2^[TRH] were indicated by single and double asterisks, respectively.

Compound	k’_IAM_	t_1/2 _(min)	Sleeping time (min)
Vehicle	N/A	N/A	80±2
[Glu^2^]TRH, R=H	0	N/A	65±3*
R=Me	0.13	20	54±1**
R=Hex	16.0	22	50±2**
R=cHex	6.02	55	55±2**
R=tBu	1.67	70	58±1*
R=Bz	5.48	>120	72±2

## 3. Prodrug-amenable analogues of TRH and related peptides as CNS Agents

As we have shown above, [Leu^2^]TRH was successfully transported into the brain, albeit with a rather complex prodrug approach that required not only cumbersome synthetic procedures [25], but also a series of enzymatic processes for liberating the parent peptide. Our investigations have uncovered shortcomings of this strategy presented schematically in Figure 2. The most serious of them was that these types of prodrugs are extremely lipophilic (calculated logP=6.20) and, thus, essentially water-insoluble. Therefore, organic solvents or pharmaceutical excipients (e.g., 2-hydroxypropyl-b-cyclodextrin) had to be used for making experimental formulation to complete *in vivo* studies. The high likelihood that these complex constructs would elude successful pharmaceutical development was also indicated by their complete non-compliance with Lipinksi’s rule-of-five [59] that generates alert for compounds where two of the following conditions are satisfied: molecular weight >500 Da, number of H-bond donors >5, number of H-bond acceptors >10, and calculated logP >5 or Moriguchi’s MlogP >4.15 [60]. Although not without limits, the Lipinksi’s rule-of-five is generally applied as one of the best criteria for drug-likeness of a compound. Therefore, we have turned our attention to an alternative approach to solve these problems, while keeping the benefits of the dihydropyridine-based brain-targeting approach [26,27]. 

### 3.1. TRH analogues by replacement of the basic central residue

**Figure 4:**
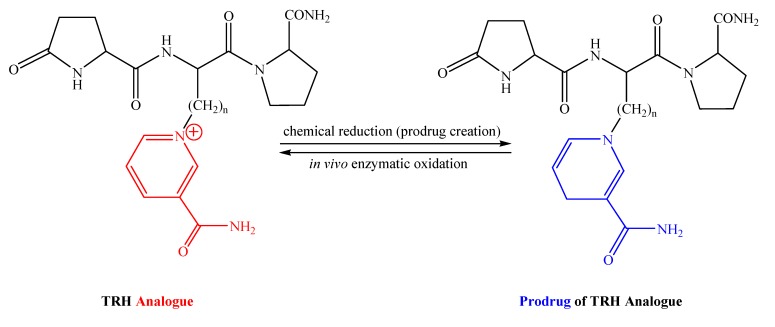
Prodrug-amenable [pyridinium^2^]TRH analogues and their corresponding brain-targeting prodrugs (n=1-4).

To utilize the dihydropyridine-containing transport moiety for peptide-based brain delivery, one has to first design/discover specific analogues/mimetics of the target peptide that contain pyridinium residues suitable for chemical reduction, thus, prodrug creation. As such, first we replaced the basic central His (containing the ionizable imidazole ring), believed to be an essential structural element to the full thyrotropin-releasing activity but not to CNS-effects [61], with pyridinium derivatives resulting in novel TRH analogues (Figure 4) that are directly amenable for brain-targeting prodrug creation [62,63] by simply reducing the pyridinium moiety to the neutral dihydropyridine. We also hypothesized that a permanently charged pyridinium moiety directly replacing the ionizable imidazole ring of His would yield the closest analogue (n=1, Figure 4) of TRH and, thus, expected to endow the maximum CNS-activity. 

This hypothesis was tested by the synthesis and comparative evaluation of additional side-chain elongated homologues (n=2–4). The TRH analogues were prepared by semi-automated solid-phase peptide synthesis utilizing fluorenylmethyloxycarbonyl chemistry and benzotriazole-1-yl-oxy-tris-pyrrolidino-phosphonium hexafluorophosphate/*N*-hydroxybenztriazole/diisopropylethylamine coupling [60]. Introduction of the pyridinium moiety was also carried out by solid-phase Zincke-reaction [64]. The corresponding prodrugs were obtained upon straightforward reduction of the central pyridinium to dihydropyridine with Na_2_S_2_O_4_ [50] or NaBH_4_ [62]. These neutral brain-targeting prodrugs apparently reach the brain where, after enzymatic oxidation (from dihydropyridine to pyridinium [26]) the analogues are regenerated at the site of action. A measurement for the increase in the ability of prodrugs generated from the designed analogues was, again, evaluated by IAMC. As shown in Table 3, the dihydropyridine moiety in the prodrugs, indeed, improved the affinity of the compounds to membrane lipids when compared to the parent (ionic) analogue. 

**Table 3:** IAMC capacity factors (k’_IAM_) and computed logarithm of octanol/water partitioning (log P) as predictors for membrane permeability for TRH, its novel analogues having pyridinium central residue and their corresponding prodrugs shown in Figure 4. Adapted with permission from [63]. ©2004 American Chemical Society.

Compound	k’_IAM_	Computed logP		
		Fragment-basedmethod [65]	Quantum chem. approach [66]	Volume-based method [67]
**TRH**	0.51	-4.37	-0.27	-1.74
**Pyridinium Analogue, n=1**	0.30	-2.67	-2.15	-2.64
**Prodrug of Analogue, n=1**	0.55	-4.38	-3.14	-3.29
**Pyridinium Analogue, n=2**	0.42	-2.61	-1.85	-2.18
**Prodrug of Analogue, n=2**	0.80	-4.33	-2.92	-2.84
**Pyridinium Analogue, n=3**	0.49	-2.16	-2.67	-1.74
**Prodrug of Analogue, n=3**	1.47	-3.88	-3.30	-2.39
**Pyridinium Analogue, n=4**	0.60	-1.77	-2.68	-1.29
**Prodrug of Analogue, n=4**	2.30	-3.48	-3.55	-1.94

Interestingly, the prodrug of the analogue having the shortest side-chain (n=1) was predicted isolipophilic with TRH. On the other hand, while the measured k’_IAM_ values show the expected trend, the *in silico* predictions for logP values [based on (a) group fragments [65]; (b) semi-empirical (AM1) quantum chemical method [66]; (c) molecular volume plus H-bonding [67], respectively, displayed a great array of unrealistic discrepancies within and among the methods employed (Table 3). Clearly, an experimental validation of such predicted logP values appears to be necessary.

**Figure 5:**
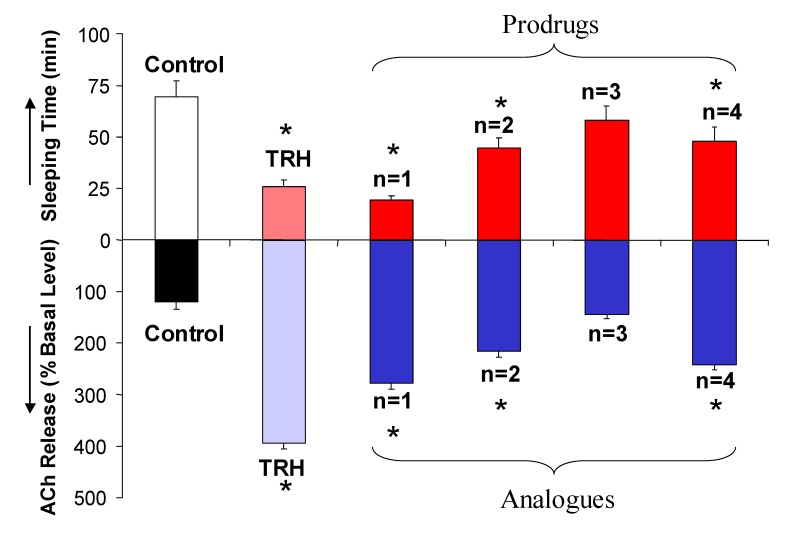
Parallel trends between the analeptic potency (i.v. injection at equimolar dose of 15 µmol/kg body weight as prodrugs) and ACh-releasing effect (perfusion of the analogues into the hippocampus by microdialysis at equimolar concentrations of 1 nmol/µl) of the novel [pyridinium^2^]TRH analogues shown in Figure 4 together with those of TRH and control (saline vehicle), respectively. * *P*<0.05 vs. vehicle control. Reprinted with permission from [65]. ©2004 American Chemical Society.

In mouse plasma and brain homogenate (20%, w/v), the prodrugs converted to the corresponding analogues with t_1/2_ around 20 min and 6 min, respectively. On the other hand, we measured a half-life of 16 min in brain homogenate and 11 min in plasma, respectively, for TRH, while all novel analogues (n=1–4) were very stable in these tissues (less than 10% degradation in 2 hours). It should be noted that the longer t_1/2_ of prodrugs in plasma compared to that of in the brain is beneficial to the CNS-sequestration of the analogues after systemic administration. Replacement of [His^2^] practically abolished binding affinity (>100 μM compared to 42 nM for TRH) to the receptor labeled by [3H][3-Me-His2]TRH in pellets obtained from rat brain Accordingly, no endocrine effect may be expected from the designed analogues having permanently charged central residue [61]. 

Initial neuropharmacological validation of our design was done by monitoring the already mentioned antagonism of barbiturate-induced anesthesia upon i.v. administration of the corresponding prodrugs (Figure 5). The saline vehicle alone (1.5 ml/kg body weight) or equimolar doses (15 µmol/kg body weight) of test compounds were injected through the tail vein. After 10 min, each animal received an i.p. injection of sodium pentobarbital at a dose of 60 mg/kg. A statistically significant (p<0.05) decrease in the sleeping time was achieved by the administration of prodrugs of analogues having n =1, 2 and 4 side-chains, compared to the control. Moreover, the analogue with the shortest side-chain (n=1) produced TRH–equivalent analeptic action when administered in its prodrug form. In that regard, our hypothesis was correct in terms of predicting that the permanently charged pyridinium moiety directly replacing the ionizable imidazole ring of His would yield the closest analogue of TRH and, thus, produce maximum CNS-activity among the homologues. As expected, without neutral prodrug creation, the ionic analogues were ineffective due to their inability to reach the CNS when administered by parenteral injection (data not shown).

TRH and related peptide are apparently exerts their analeptic action thorough cholinergic mechanism [36,37]. Therefore, in another set of experiments, we measured the changes in hippocampal acetylcholine (ACh) concentration when the analogues (n=1–4, Figure 4) were perfused through a microdialysis probe placed into the hippocampus of rats (Figure 5) [68]. Although none of the analogues outperformed TRH in this experimental model at equimolar concentration (1 nmol/µL), they significantly increased ACh levels compared to the control. Even the most potent analogue (n=1) exerted an approximately 30% lower stimulation of the ACh release compared to that of TRH. The increase in the length of the side-chain (from n=1 to n=4) showed a similar tendency observed for the analeptic action. Again, ACh production levels after administration of analogue with n=3 were not significantly different from those of obtained after perfusion of the control only, given the error calculated from the experiments. Figure 5 also shows a strong connection between the analeptic activity and the ACh-releasing capacity of TRH analogues and TRH, respectively. Elongation of the side-chain implied a V-shape tendency in producing the pharmacological responses among the homologues. This trend remains to be explored and, perhaps, exploited in a future study. 

**Figure 6:**
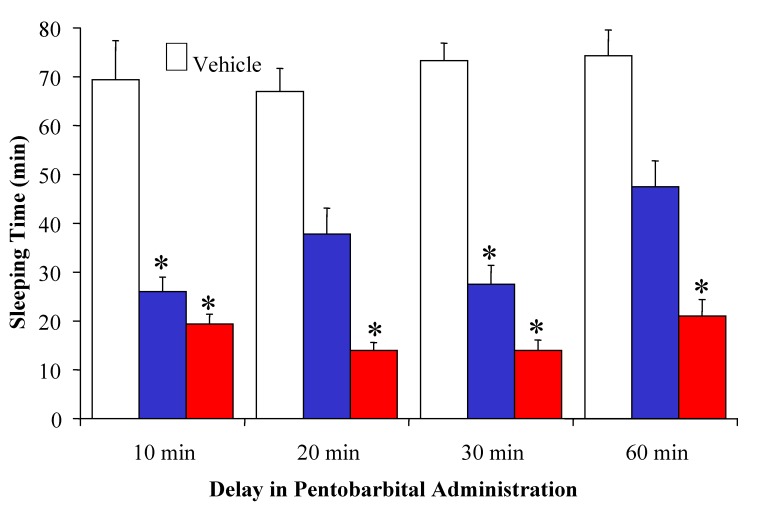
Comparison of the analeptic effect of equimolar doses (15 µmol/kg body weight, i.v.) of TRH (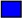 ) and its [pyridinium^2^] analogue with n=1 (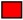 ) administered in its prodrug form upon varying the time for pentobarbital treatment (60 mg/kg body weight, i.p.) post-drug administration. * indicates statistically significant differences (ANOVA followed by Dunnett’s test, *P*<0.05) from vehicle control. Reprinted with permission from [63]. ©2004 American Chemical Society.

The most promising analogue (n=1) was further studied and compared to TRH for the duration of the analeptic action by increasing the time after which the cholinergic challenge was made in the animals injected with TRH or the prodrug of this analogue (Figure 6). The analeptic activity of TRH showed an overall decrease, when the time between the injections was increased. A loss of over half of CNS-activity was observed at 60 min, compared to the value measured when the pentobarbital injection was made 10 min after TRH exposure to the mice. Administration of the prodrug, however, produced the shortest sleeping time, when the cholinergic challenge was done 20 to 30 min post drug administration. Moreover, the analeptic effect still remained comparable to the maximum value reached by TRH in our experiments even 60 min after administration of the pentobarbital. This sustained CNS effect may be attributed to the metabolic stability of the designed analogue.

**Figure 7:**
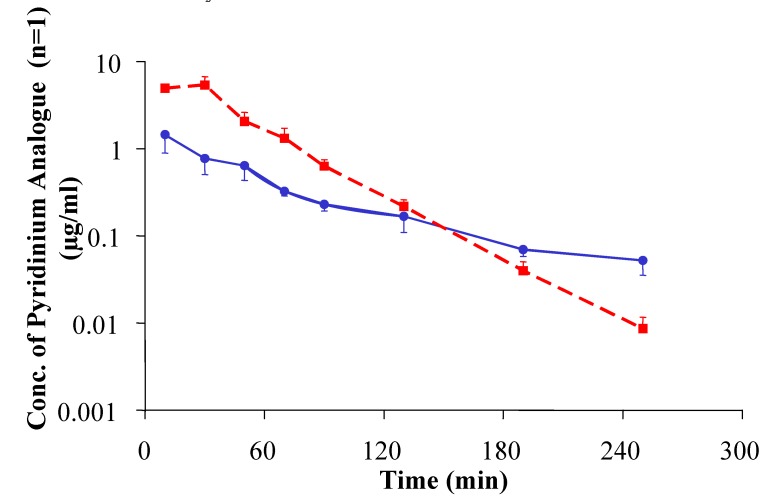
Concentration of the [pyridinium^2^]TRH analogue with n=1 (i.p. injection of its prodrug at a dose of 15 mg/kg body weight) in the hippocampus (circles, blue solid line) and in blood (squares, red dashed line) of rats, measured via in vivo microdialysis sampling followed by LC/ESI-MS/MS assay. Reprinted with permission from [63]. ©2004 American Chemical Society

This observation was in good agreement with the pharmacokinetics (PK) and brain uptake/retention profile expected for and actually manifested by the prodrug approach, according to the concentrations measured in the brain and blood for the most potent analogue (n=1) after the i.p. injection of its prodrug to rats. To obtain the PK and brain uptake/retention profiles, we used *in vivo* microdialysis to continuously and simultaneously collect samples from the extracellular space of the hippocampus and from the jugular vein. High-performance liquid chromatography coupled with electrospray ionization tandem mass spectrometry (HPLC/ESI-MS/MS) [58] was used to measure the concentrations of the analogue (n=1) in the hippocampus and in the blood. As shown in Figure 7, both the brain and blood concentrations of the TRH analogue reached its maximum within 30–50 min after drug administration. The measured highest extracellular level of the analogue in the hippocampus was about 3–4-times lower than its concentration in the blood; the first-order rate constant for its subsequent elimination from the systemic circulation was, however, about 3-times higher (1.62 h^-1^) than from the specified CNS location (0.59 h^-1^). Therefore, the concentration of this TRH analogue in the hippocampus and in the blood became equal around 120–150 min, and the CNS level of this compound was approximately 6-times higher than its concentration in the systemic circulation 4 hours after i.p. administration of its prodrug. 

Overall, we consider this agent having a central pyridinium residue (n=1, Figure 4) as a novel type of prodrug-amenable lead for exclusively centrally acting and metabolically stable TRH analogues. It is also noteworthy that the prodrugs of the [pyridnium^2^]analogues, unlike the very lipophilic CNS-targeting prodrug of [Leu^2^]TRH shown in Figure 2, can easily be formulated in an aqueous vehicle. In addition, they do not raise alert when subjected to Lipinski’s rule-of-five [59].

### 3.2. [Glu^2^]TRH analogues with pGlu-modification strategy

**Figure 8:**
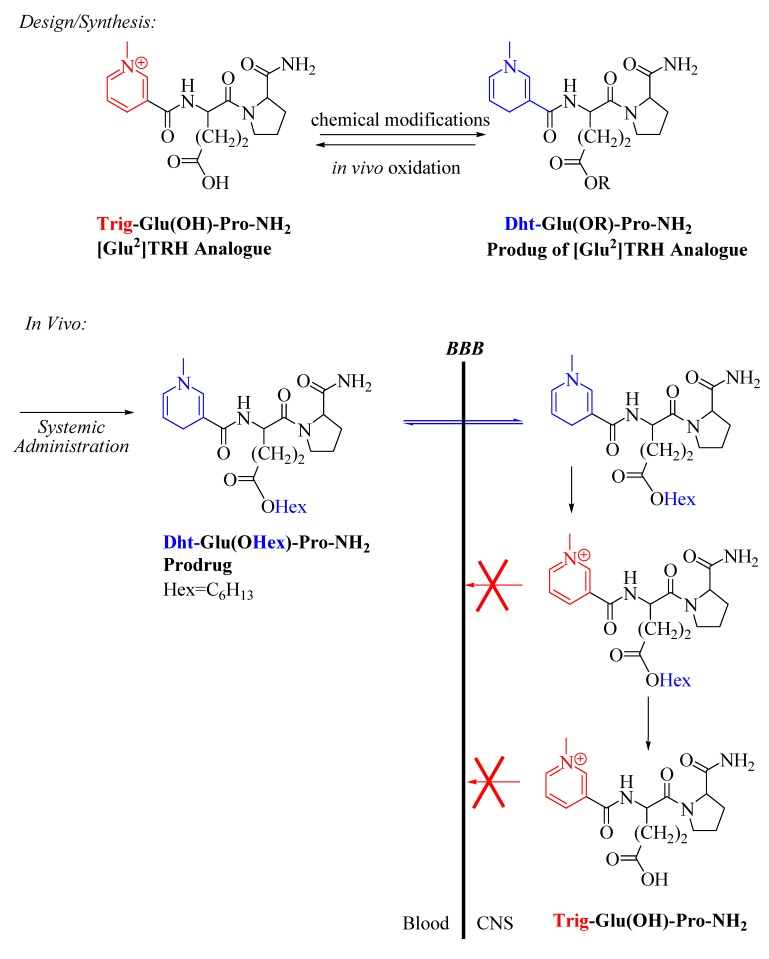
Schematic illustration of the brain delivery concept for a [Glu^2^]TRH analogue, Trig-Glu-Pro-NH_2_, by prodrug design.

After evaluating the promising, exclusively CNS-active TRH analogue shown in Figure 4, we became interested in probing whether a prodrug-amenable pyridinium analogue retaining or perhaps selectively enhancing certain CNS-actions could also be obtained from [Glu^2^]TRH as a lead compound. For the latter tripeptide, we decided to substitute its N-terminal pGlu because the therapeutically most successful analogues have been derived via such a modification of TRH. For example, 1-methyl-(*S*)-4,5-dihydroorotyl-l-histidyl-l-prolinamide (taltirelin, TA-0910) has received approval (Ceredist®, Japan) for the treatment of adult spinal muscular atrophy [69]. Additionally, we had also confirmed that aliphatic ester prodrugs (especially Hex) significantly improved CNS targeting of this peptide (Table 2). Therefore, we decided to keep the Glu-Pro-NH_2_ motif and replace this time the amino-terminus (pGlu) with a pyridinium-based (Trig) residue and explore the potential of this compound as a CNS-agent (Figure 8) [70]. The presence of Trig and γ-carboxylic acid of Glu in the molecule allowed us for bioreversible “double lipophilization” to obtain (pro)-prodrugs suitable for BBB transport. Analogously to that of design shown in Figure 4, once the prodrugs diffused through the BBB, the dihydropyridine (Dht) undergoes enzymatic oxidation [26] and produces the cationic compounds (with or without the ester group on the side-chain of Glu, depending on the rate of its hydrolysis) whose efflux from the brain is, therefore, prevented (Figure 8). 

Using IAMC, we confirmed that although the neutral prodrug, without the ester function on the side-chain of the central residue (Dht-Glu(OH)-Pro-NH_2_), improved the affinity of the prodrug to membrane lipids compared to the ionic analogue by approximately three- to five-fold (k’_IAM_ = 0.4); however, it still showed poor affinity to IAMC. Therefore, a systematic variation of the ester moieties was undertaken that allowed us, again, to find/confirm the optimum for this promoiety to modify the side chain of the Glu residue and yield efficacious prodrugs of Trig-Glu-Pro-NH_2_ (Figure 8). *In vitro* stability studies indicated that the oxidation of the neutral prodrug (Dht-Glu(OR)-Pro-NH_2_) to the ionic counterpart occurred faster in mouse brain homogenate (t_1/2_=6 min) than in heparinized mouse blood (t_1/2_=16 min) that is favorable for promoting CNS-retention. On the other hand, a slower hydrolysis of the ester in the mouse brain homogenate (e.g., t_1/2_=27 min for the Hex ester) implied a sustained formation of the [Glu^2^]TRH analogue depicted in Figure 8 in the brain and thus, long-lasting CNS-mediated pharmacological effects may be expected. Additionally, the metabolic stability (<10% degradation in 2h) of the analogue (Trig-Glu(OH)-Pro-NH_2_)was also confirmed. 

The analeptic action of Trig-Glu(OH)-Pro-NH_2_ (Figure 8) was used to optimize the efficacy of this potential CNS agent when administered i.v. in its CNS-permeable prodrug forms (Dht-Glu(OR)-Pro-NH_2_) obtained *via* reduction of the pyridinium moiety (Trig) to the neutral dihydropyridine (Dht) and esterifying the central Glu with various alcohols (R-OH) [70]. The maximum effect in antagonizing pentobarbital-induced narcosis in mice (18 ± 2 % decrease compared to that of saline control counted as 100%) was, again, achieved with the administration of the hexyl ester prodrug, Dht-Glu(OHex)-Pro-NH_2_ (Table 4). This prodrug also showed quite high k’_IAM_ value (28.6). It is noteworthy, that the hexyl ester prodrug of the parent [Glu^2^]TRH was approximately twice as potent in reversing the effect of the cholinergic challenge than that of the analogous prodrug of Trig-Glu(OH)-Pro-NH_2_. As such, the latter evoked practically the same sleeping time as the unmodified [Glu^2^]TRH *per se* (Table 4). As proof-of-concept, we also tested Trig-Glu(OHex)-Pro-NH_2_ (the hexyl ester of the designed [Trig^1^] analogue of [Glu^2^]TRH) without reducing Trig to Dht and, thus, rendering the molecule neutral. As expected, no pharmacological response was observed with this ionic compound due to its inability to diffuse through the BBB. 

**Table 4:** Comparison of analeptic (equimolar dose of 10 µmol/kg body weight, i.v.) and antidepressant (equimolar dose of 3 of µmol/kg body weight, i.v) effects of test compounds in mice. Values are expressed as percent decrease compared to those of saline vehicle that was counted as 100%. *Statistically significant difference from saline control (ANOVA followed by Dunnett’s, *P* < 0.05, n=6–16). Reprinted with permission from [70], ©2005 Bentham Science.

Compound	Analeptic Effect:% Decrease in Sleeping Time	Porsolt Swim Test:% Decrease in Immobility
**TRH: pGlu-His-Pro-NH_2_**	50±2*	36±2*
**Glu^2^[TRH]: pGlu-Glu-Pro-NH_2_**	17±2*	33±4*
**pGlu-Glu(OHex)–Pro-NH_2_**	37±3*	44±2*
**Dht-Glu(OHex)–Pro-NH_2_**	18±2*	43±3*
**Trig-Glu(OHex)–Pro-NH_2_**	1±3	2±1

We selected the “best-performing” Dht-Glu(OHex)-Pro-NH_2_ prodrug in the above experimental paradigm for a subsequent comparative neuropharmacological evaluation with pGlu-Glu(OHex)-Pro-NH_2_, the hexyl ester prodrug of the parent [Glu^2^]TRH (Figure 3). Specifically, we employed the Porsolt swim test (PST) [70], an established behavioral paradigm to screen compounds for antidepressant activity and potency in rodents (Table 4). TRH and related peptides are well known to possess antidepressant effect [29,32]. In PST, we used mice that are put individually in an inescapable cylinder of water for a given period of time, and the time (duration) that they swim until remain floating (immobile) is measured. Immobility is considered to reflect a "depressive mood." In the control group, animals are injected with saline only. Reduction in the duration of immobility time by the test compounds compared to that of saline-injected animals is considered an antidepressant-like effect. Table 4 shows that administration of Dht-Glu(OHex)-Pro-NH_2_ (3 µmol/kg body weight, i.v.) afforded significant reduction in the immobility time—identical to that of pGlu-Glu(OHex)-Pro-NH_2_, the “best-performing” [Glu^2^]TRH prodrug in regard of evoking analeptic effect compared to the saline control (Table 2). At the same time, it is noteworthy that the analeptic effect of the latter was significantly (by 200%) higher. Therefore, this observation has indicated that the analeptic and antidepressant activity can be dissociated. Additionally, both prodrugs outperformed TRH in the test employed. As expected, no pharmacological effect was recorded in the PST without rendering the hexyl ester of the [Trig^1^] analogue “neutral” and, thus, eliminating the permanent positive charge by reducing Trig to Dht, in spite of the significantly increased lipid-membrane affinity. Taken together, Trig-Glu(OH)-Pro-NH_2_ may represent a centrally active TRH analogue with improved selectivity of its CNS-action and a lead for a novel type of antidepressants.

## 4. Conclusions

The pleiotropic action of TRH in the CNS makes this neuropeptide an attractive template for drug design and discovery. However, several limitations of TRH-based neurotherapy need to be addressed, including metabolic instability, poor CNS bioavaibility and selectivity. Because TRH *per se* also is an endocrine hormone, drug discovery efforts involving the CNS have focused thus far on designing TRH analogues/mimetics that have diminished, if any, hormonal effect and show sufficient resistance against proteolytic degradation in the blood. However, delivery of these agents into the CNS has remained a crucial aspect of the drug discovery process. For this purpose, we promote the prodrug approach as a versatile and bioreversible chemical manipulation technique that furnishes the target agent with adequate lipophilicity necessary for passive transport through the BBB. When coordinated with the discovery and development of prodrug-amenable analogues, this approach may provide specific molecular probes by which the central action of TRH can be studied by using convenient systemic administration, and it also offers potential treatment for various CNS maladies.
